# PTSD, dissociative experiences, and depressive symptoms in a clinical sample of women who featured in pornographic productions

**DOI:** 10.1192/j.eurpsy.2024.638

**Published:** 2024-08-27

**Authors:** F. K. El Khoury, V. Héroin, M. Fareng

**Affiliations:** ^1^Department of social epidemiology, Sorbonne Université, INSERM, IPLESP; ^2^Clinical Psychologist, Paris, France

## Abstract

**Introduction:**

The mental health of the people who featured in Pornographic Productions (PP) is underexamined. However, PP frequently involve unsimulated violent acts mostly experienced by women. Furthermore, some women participating in PP also report being coerced into unwanted sexual acts. Therefore, featuring in a PP could be experienced as a traumatic event, and could be associated with negative mental health disorders.

**Objectives:**

Our study examines mental health indicators among Women who have participated in at least one PP (WPP), and who consulted clinical psychologists, after referral by WPP support groups.

**Methods:**

Thirty-six women were recruited by two clinical psychologists during an individual consultation. Participants completed the French versions of the post-traumatic stress disorder (PTSD) Checklist for DSM-5 (PCL-5), the Dissociative Experiences Scale (DES), as well as the 13-item Beck Depression Inventory (BDI-13). Data on socio-demographic characteristics, lifetime experience of sexual violence prior to participating in a PP, as well as the perceived effect of participating in a PP were also measured.

**Results:**

The mean age of participants was 31.2 (std=7), and the average age at first participation in a PP was 23.4 (std=6). The majority (78%) of participants reported lifetime experience of sexual violence prior to participation in a PP. Thirty women (83%) had a PCL5 score over 33 indicative of probable PTSD, and 28 women (78%) had a DES score of 30 or more indicating high levels of dissociation. Further, 16 participants (44%) reported a BDI-13 score over 16 indicating severe depression.

**Conclusions:**

This study highlights the high prevalence of PTSD, dissociative experiences, and depressive symptoms in a clinical population of women who featured in at least one PP. Further studies are needed to better understand the scale of the problem and optimize care interventions.

**Disclosure of Interest:**

None Declared

